# Peptidomics Profile, Bioactive Peptides Identification and Biological Activities of Six Different Cheese Varieties

**DOI:** 10.3390/biology12010078

**Published:** 2023-01-02

**Authors:** Ahmed Helal, Davide Tagliazucchi

**Affiliations:** 1 Department of Food and Dairy Sciences and Technology, Damanhour University, Damanhour 22516, Egypt; 2 Department of Life Sciences, University of Modena and Reggio Emilia, Via Amendola, 2-Pad. Besta, 42100 Reggio Emilia, Italy

**Keywords:** mass spectrometry, peptide profiling, proteomics, dairy, biological activity

## Abstract

**Simple Summary:**

The consumption of cheese is increasing from year to year, as this food is considered rich in nutrients, calcium and vitamins. Recently, cheese consumption has been associated with a lower incidence of cardiovascular diseases and type-2 diabetes. In this work, we characterized three traditional Egyptian cheeses (Karish, Domiati and Ras) and three additional cheese varieties consumed worldwide (Feta-type, Gouda and Edam) for their content of bioactive peptides (the main bioactive component of cheese responsible for its health benefits) and for their biological activities. Most of the identified bioactive peptides were able to inhibit key enzymes involved in the progression of cardiovascular diseases (angiotensin-converting enzyme, ACE) and diabetes (di-peptidyl-peptidase-IV, DPP-IV). Two well-known and studied bioactive peptides with anti-hypertensive activity in humans, called VPP and IPP, were quantified in the different cheeses. The results showed that the consumption of 10–20 g of Gouda cheese, 50–100 g of Domiati and 100 g of Edam should be enough to exert anti-hypertensive activity. Moreover, Gouda and Edam cheeses also contained a high amount of the DPP-IV-inhibitory peptide APFPE, suggesting a possible role in the prevention of diabetic complications.

**Abstract:**

Several recent published studies reported that cheese consumption may protect against the onset of cardiovascular diseases and type-2 diabetes due to the presence of bioactive peptides. In the present work, six cheese varieties (the Egyptian traditional cheeses Karish, Domiati and Ras as well as Feta-type, Gouda and Edam cheeses) were characterized for their peptidomics profiles with high-resolution mass spectrometry, biological activities and content in bioactive peptides. The highest ACE-inhibitory and DPP-IV-inhibitory activities were found in Gouda cheese, which also displayed the highest antioxidant activity. A total of 809 peptides originating from the major milk proteins were identified, and 82 of them were bioactive. Most of them showed ACE-inhibitory, antioxidant and DPP-IV-inhibitory activities. The highest amount of the in vivo anti-hypertensive tripeptides VPP and IPP was found in Gouda cheese (39.19 ± 1.26 and 17.72 ± 0.89 mg/100 g of cheese, respectively), whereas the highest amount of APFPE was detected in Edam cheese (509.13 ± 20.44 mg/100 g of cheese). These results suggest that the intake of Edam, Domiati and, especially, Gouda cheeses may result in a possible anti-hypertensive effect in hypertensive subjects.

## 1. Introduction

Cheese is one the most consumed dairy products worldwide and is produced from casein coagulation and precipitation following acidification or fermentation. The per capita consumption of cheese, and dairy products in general, has continuously increased in the last years and is predicted to further increase by approximately 14% in the next 10 years [1]. Overall, approximately 2000 cheese types have been documented that differ by the milk used, the coagulation and fermentation processes as well as the ripening time [1]. Most cheeses are ripened for a period ranging from a few weeks to two or more years; however, some cheeses are not aged for a significant amount of time and are classified as fresh cheeses [1,2].

Cheeses are rich in nutrients, such as proteins, fatty acids, minerals (in particular calcium) and several vitamins (especially vitamins A and B_12_), but they also contain bioactive molecules, most of them coming from the metabolism of lactic acid bacteria [3,4,5]. Due to the high content of bioactive compounds, it is not surprising that cheese has been the subject of several in vivo studies and meta-analyses. Most of the studies focused on cheese consumption and cardiovascular diseases. For example, the intake of a standard serving of cheese (50 g) resulted in a 10–14% reduction in the risk of coronary heart disease, whereas a meta-analysis study revealed a 6% lower risk of stroke in subjects consuming a high amount of cheese in comparison with low consumption [6,7]. Moreover, a case-cohort study involving eight European countries suggested a possible inverse relationship between cheese consumption and type-2 diabetes [8]. In addition, cheese consumption may also affect gut microbiota composition. For example, Firmesse et al. [9] demonstrated that the microorganisms in Camembert cheese were able to colonize the human colon in healthy volunteers. More recently, Aljutaily et al. [10] showed that the intake of a probiotic cow cheese caused changes in the gut microbiota composition that resulted in a healthier gut microbiota compared to a standard diet.

Among the different bioactive compounds present in cheese, particular attention has been paid in the last years to bioactive peptides [4,5]. They are defined as short amino acid sequences present in milk proteins that, once released from the parent protein by the action of proteases, may have a positive impact on human physiology and health [11]. The hydrolysis of milk caseins begins during the early phases of cheese production due to the action of chymosin; endogenous proteases, such as plasmin and somatic cell proteolytic enzymes; as well as proteases in starter lactic acid bacteria [2]. These initial events generate large or medium oligopeptides that are further cleaved into short peptides by the action of cell-envelope proteases and the peptidases of nonstarter lactic acid bacteria during cheese maturation and ripening [12].

Numerous bioactive peptides have been reported in both fresh and ripened cheeses, potentially exerting a plethora of biological activities. Most of the bioactive peptides identified in cheese are angiotensin-converting enzyme (ACE) inhibitory and anti-hypertensive peptides, though other peptides with antimicrobial, anti-diabetic, immunomodulatory, opioid, mineral-binding and antioxidant activities have been identified [3,5,12]. In the last decade, bioactive peptides have been identified in various commercial cheeses manufactured with different technologies and ripening times, such as Parmigiano Reggiano, Prato, Grana Padana, Gouda and Cheddar, as well as in various kinds of Spanish, Turkish and Swiss cheeses [13,14,15,16,17,18,19,20]. Some of the peptides identified in cheeses are proven to have biological activity in humans, such as the anti-hypertensive peptides VPP and IPP, or in rats, such as the anti-hypertensive peptides YP, KVLPVPQ, LHLPLP and the anti-diabetic peptide LPQNIPPL [12]. The presence and the amounts of bioactive peptides in cheeses depend on several factors, such as the production process, the ripening time, the starter lactic acid bacteria and the peculiar microbiota that colonizes cheese during ripening [5].

There are many different cheeses produced in Egypt; most of them have never been characterized for their peptidomics and biological activity profiles.

Karish, Ras and Domiati are some examples of cheeses produced in Egypt. Ras cheese (also known as Roumy cheese) is the most popular hard cheese in Egypt made with raw cow milk and added rennet, cooked for 45 min at 45 °C and ripened up to 3 months. On the other hand, Domiati is a soft cheese also manufactured from raw milk with added rennet but not cooked and then ripened up to 3 months. Both of these cheeses are produced without adding starter cultures. On the contrary, Karish cheese is a fresh non-ripened cheese manufactured with pasteurized buffalo skimmed milk inoculated with a starter culture of lactic acid bacteria. Gouda and Edam cheeses are consumed worldwide, originally from the Netherlands, but they are also produced in Egypt. Both of these cheeses are made from pasteurized cow milk with the addition of starter cultures and cooked and ripened up to 3 months. Feta-type cheese is another cheese consumed worldwide and is one of the most popular in Egypt. It is a fresh cheese manufactured with pasteurized skimmed cow milk and subjected to ultrafiltration to concentrate the proteins with added rennet. In this type of cheese, the milk fats are substituted with palm oil.

The objective of this study was to define the peptidomic profiles of six different cheese varieties with high-resolution mass spectrometry and characterize the peptide fractions for their biological activities (antioxidant, ACE-inhibitory and dipeptidyl-peptidase IV-inhibitory activities). Next, an in silico analysis was carried out to identify the bioactive peptides in the cheeses.

## 2. Materials and Methods

### 2.1. Materials

The enzymes and chemicals for the in vitro biological activity assay and for the peptide quantification were from Sigma-Aldrich (Milan, Italy), whereas the solvents for the mass spectrometry analysis were purchased from Biorad (Hercules, CA, USA). The Amicon Ultra-4 regenerated cellulose ultrafiltration units (3 kDa cut-off) were obtained from Millipore (Milan, Italy). All the other reagents were from Carlo Erba (Milan, Italy).

The buffalo and cow milk were obtained from a local animal farm (Al Nubaria, Egypt), whereas palm oil was purchased from the local agency (Arma, Cairo, Egypt). RENIPLUS^®^ dried microbial rennet from plant origin was from Proquiga Biotech (La Coruña, Spain). Freeze-dried lactic cultures MO-30 (containing *Lactococcus lactis* ssp. *cremoris* and *Lactococcus lactis* ssp. *Lactis*), CHN-11 (containing *Lactococcus lactis* subsp. *cremoris*, *Leuconostoc*, *Lactococcus lactis* subsp. *lactis* and *Lactococcus lactis* subsp. *lactis* biovar diacetylactis) and YF-L811 (a yoghurt starter culture containing *Streptococcus thermophilus* and *Lactobacillus delbrueckii* subsp. *bulgaricus*) were obtained from CHR-Hansen Lab (Denmark). Glucono delta-lactone (GDL) was obtained from Roquette (Lille-France), whereas ANNATO 055-MF-WS, a natural water-soluble food-grade colour, was purchased from MIFAD (Cairo, Egypt).

### 2.2. Cheese Manufacturing

In this study, the most predominant popular cheeses sold in the Egyptian market, which differ mostly in composition, starter, technological characteristics and ripening time, have been examined. The selected cheeses were made in a local factory (Reefy for dairy goods, Al Nubaria, Egypt). For all the cheese types, three independent batches of milk were used in the manufacture. In each batch, 3 replicates were manufactured of each cheese.

Table 1 summarizes the main differences in the production process for the different cheeses.

#### 2.2.1. Karish Cheese

Karish cheese was made from skimmed buffalo milk as previously described by El-Zayat and Omar [21]. Briefly, buffalo skimmed milk was pasteurized at 72 °C for 15 s before cooling to 42 °C; then, the milk was inoculated with the yoghurt starter culture YF-L811 and incubated for 4–6 h at 42 °C. The curd was then transferred to a plastic mat, according to the conventional procedure, salted (2% *w*/*w*) and left overnight to allow the whey to drain. The finished cheese was placed in polyethylene bags in a 4% saline solution and stored in the refrigerator at 5 °C.

#### 2.2.2. Feta-Type Cheese

Feta-type cheese was produced from concentrated skimmed cow milk and palm oil following the method of Abed El Malek et al. [22]. Skimmed cow milk was first subjected to pasteurization at 72 °C for 15 s, cooled to 50 °C and then separated with an ANDRITZ milk separator (Graz, Austria). The process of ultrafiltration (GEA membrane filtration pilot units) was used to obtain concentrated skimmed milk with a ratio of 1:4 concentration. Palm oil was then added and homogenized with the concentrated skimmed milk using a single-stage homogenizer (GEA Niro Soavi Homogenizer, Parma, Italy) at 50 °C and 50 bar of pressure. Next, 3% NaCl and 2.5% GDL were added to the mixture and then distributed on trays, while the rennet was also added at 40 °C and left for complete coagulation and cooling. The final cheese was sliced into cubes similarly to Domiati cheese. The cheese was stored in the fridge at 5 °C in plastic containers that had 7.5% NaCl permeate in them.

#### 2.2.3. Domiati Cheese

According to Fahmi and Sharara [23], to produce Domiati cheese, cow milk was heated to 37 °C, and 8% of salt was added. After filtration through a fine muslin cloth, rennet was added and left to coagulate for 100 min. The curd was transferred into rectangular stainless-steel moulds lined with muslin cloth and then covered and lightly pressed overnight with a weight. The Domiati cheese was produced and cut into 10 cm cubes before being packed into tins lined with polyethylene bags and pickled in their own drained salty whey and stored for 90 days to ripen.

#### 2.2.4. Ras Cheese

Following the traditional method of making Ras or Roumy cheese as described by Hofi et al. [24], in a cheese vat, raw cow milk was warmed to 32 °C, and small amounts of annatto were added to enhance the colour. Rennet was then added to coagulate the milk for approximately 45 min. Later, the curd was cut, and the temperature was then raised to 45 °C for cooking over 45 min. The curd was salted using NaCl (3%, *w*/*w*) after draining a third of the whey. After 15 min, the rest of the whey was drained, and the cheese was cooled, moulded and pressed for 12 h. The cheese wheels were then subjected to dry-salt on the two surfaces and were stored for 90 days at 12–15 °C and 85% relative humidity.

#### 2.2.5. Gouda Cheese

Gouda cheese was manufactured from 3.5% fat pasteurized cow milk (65 °C/ 30 min) as described by Park et al. [25]. Calcium chloride (0.02%, *w*/*w*) was added, and then the milk was inoculated with the cheese starter culture MO-30 and CHN-11 in an amount of 5 and 2.5 units per 100 litres of milk, respectively. Annatto was also added in small amounts as a colouring agent. Rennet was added to stimulate the production of curds. The milk was left to coagulate for 45 min at 30 °C before being sliced into 7 mm cube-shaped particles and stirred for 20 min. Approximately 33% of the cheese whey was removed and replaced with hot water (approximately 55 °C), and the curds were stirred for 45 min with the end temperature being 38 °C. The whey was then drained, and the curd was mechanically squeezed in the vat. Later, the curd was placed in a mould and pressed with gradually increasing pressure (4–6 h), then left squeezed overnight. The Gouda cheese was removed from the mould and immersed in a 20 % NaCl saturated brine solution for 2–3 days at 12–15 °C. The ripening process was carried out for 90 days at a temperature of 12–15 °C and a relative humidity of 85%.

#### 2.2.6. Edam Cheese

Edam cheese was produced as described in Aljewicz et al. [26] with some modifications. Briefly, the standardized milk (2.5% fat) was pasteurized (65 °C/30 min), and the starter cultures for the cheese, MO-30 and CHN-11, were added in an amount of 5 and 2.5 units per 100 litres, respectively. Furthermore, annatto was used as a colouring agent at low concentrations. Calcium chloride (0.02%) was used after the starter addition to optimize renneting. In conjunction with the addition of the starter culture, the milk coagulation process (approximately 30 °C) occurred and was aided by the activity of the renneting enzymes. The produced curd was then sliced approximately 40 min after the rennet addition. After that, the curd was stirred gently for 20 min. Then, half of the whey was drained and replaced with hot water (55–60 °C), bringing the temperature of the curd–whey mixture to 36 °C, where it stayed for 40 min. The whey was withdrawn, and the curd was mechanically squeezed in the vat to assist it to fuse together. The curd was then placed in moulds, and the light pressure was then gradually increased and left overnight. The squeezed curd was immersed in saturated brine (20% NaCl by weight) at 12–15 °C for two to three days. The Edam cheese was then placed in ripening cellars for 90 days (12–15 °C with 85% relative humidity).

### 2.3. Cheese Compositional Analysis

The different cheese samples were analysed for their proximate parameters (moisture, fat and protein) by using the Association of Official Analytical Chemists [27] methods. The total protein content was calculated as nitrogen and multiplied by 6.38. The salt content was determined using the Volhard method [28].

### 2.4. Extraction and Quantification of Low-Molecular Weight Peptides from Cheese Samples

The extraction of the low-molecular weight peptides from the cheese samples was carried out as reported in Martini et al. [29]. Initially, 5 g of cheese was mixed with 45 mL of 0.1 mol/L HCl, and the mixture was then homogenized by using an Ultra-Turrax homogenizer for a total of three cycles of 1 min each, interspersed with 1 min of rest on ice. The homogenized samples were then centrifuged for 40 min (4000× *g*; 4 °C), and the supernatant was filtered with Whatman filter paper 4.

Thereafter, the peptide fractions were ultrafiltered with a cut-off of 3 kDa to obtain the low-molecular weight peptide fractions as reported in Tagliazucchi et al. [30]. The extraction procedure was performed in triplicate, and the low-molecular weight peptide fractions were combined before the analysis.

The amount of low-molecular weight peptides was determined by using the TNBS (2,4,6-trinitrobenzenesulfonic acid) assay according to Adler–Nissen [31] with leucine as the reference standard compound. The data were expressed as mmol of leucine equivalent/100 g of cheese.

### 2.5. Biological Activity Assays

#### 2.5.1. Antioxidant Activity Determination

The antioxidant activity of the low-molecular weight peptide fractions of the different cheeses was assessed with two different assays. The radical scavenging activity was determined by using the ABTS assay as described in Re et al. [32], whereas the reducing ability was ascertained as reported in Benzie and Strain [33]. In both assays, ascorbic acid was used as the reference compounds, and the data were expressed as mg of ascorbic acid equivalent/100 g of cheese.

#### 2.5.2. Assessment of Inhibitory Activity against α-Amylase and α-Glucosidase

The inhibitory ability of the low-molecular weight peptide fractions against α-amylase was determined as described in Cattivelli et al. [34]. Briefly, 45 μL of different concentrations of low-molecular weight peptide fractions were pre-incubated for 20 min at 37 °C with 5 μL of porcine α-amylase solution (2 U/mL). At the end of the pre-incubation, 50 µL of starch solution (1% starch in 0.1 mol/L pH 6.9 sodium phosphate buffer containing 6.7 mmol/L of NaCl) was mixed with the reaction mixture, and the reaction was carried out for a further 10 min at 37 °C. The breakdown products of starch were quantified by mixing the reaction mixture with 50 µL of dinitrosalicylic acid solution and boiling for 15 min. After boiling, 450 µL of distilled water was added to the mixture. The absorbance was read by using a microplate reader at 540 nm after transferring 200 μL of the solution to a 96-well plate. The results were expressed as the IC_50_ values calculated by non-linear regression analysis plotting the peptide concentration (expressed as base-10 logarithm) versus the percentage of α-amylase inhibition. The IC_50_ values were expressed as mg of cheese/mL.

The inhibitory ability of the low-molecular weight peptide fractions against α-glucosidase was determined as described in Martini et al. [29]. For the assay, 20 µL of different concentrations of low-molecular weight peptide fractions were mixed with 66.7 µL of potassium phosphate buffer (67 mmol/L, pH 6.8), 5 µL of 0.2 U/mL yeast α-glucosidase and 3.3 µL of 3 mmol/L glutathione. The mix was pre-incubated for 20 min at 37 °C before the addition of 5 µL of the substrate (*p*-nitrophenyl-glucose 5 mmol/L). The reaction was carried out for a further 20 min at 37 °C before stopping it by adding 150 µL of 100 mmol/L of Na_2_CO_3_. The quantity of released *p*-nitrophenol was measured by reading at 405 nm with a microplate reader.

#### 2.5.3. Assessment of Inhibitory Activity against Dipeptidyl-Peptidase-IV (DPP-IV)

The capacity of the low-molecular weight peptide fractions to inhibit the enzyme DPP-IV was determined as previously described [35]. DPP-IV enzyme was extracted from rat intestinal acetone powder in Tris-HCl buffer (0.1 mol/L; pH 8). For the reaction, 10 µL of 0.1 U/mL of the extracted enzyme was pre-incubated in a 96-well plate for 20 min at 37 °C with 80 µL of different concentrations of low-molecular weight peptide fractions and 205 µL of Tris-HCl buffer (0.1 mol/L; pH 8). After pre-incubation, 5 µL of 6.4 mmol/L Gly-Pro-pNA was added to the mixture, and the reaction was further carried out for 20 min at 37 °C. At the end of the reaction, the amount of released pNA was determined by reading at 405 nm with a microplate reader. The data were expressed as the IC_50_ values measured as reported in Section 2.5.2.

#### 2.5.4. Assessment of Inhibitory Activity against Angiotensin-Converting Enzyme (ACE)

The inhibitory activity of the low-molecular weight peptide fractions against ACE was determined as previously reported [36]. In the assay, 60 µL of different concentrations of low-molecular weight peptide fractions were mixed with 70 µL of the tripeptide N-[3-(2-furyl)acryloyl]-L-phenylalanyl-glycyl-glycine (FAPGG 6 mM dissolved in Tris-Cl 100 mmol/L pH 8.2 with 0.6 mol/L of NaCl) and 10 µL of enzyme (enzyme activity in the assay of 50 mU/mL). The mixture was incubated for 3 min at 37 °C, after which the absorbance was read at 340 nm with a microplate reader (T0). Next, the reaction was carried out for an additional 20 min before reading the absorbance (T20). The difference in the absorbance between T0 and T20 represented the enzymatic activity. The data were expressed as the IC_50_ values measured as reported in Section 2.5.2.

### 2.6. Peptidomics Analysis

The peptide profile of the low-molecular weight peptide fractions was assessed with high-resolution mass spectrometry analysis. The Ultra High-Performance Liquid Chromatography (UHPLC) conditions and mass spectrometry parameters are fully described in Martini et al. [29]. The peptide identification was performed with the MASCOT (Matrix Science, Boston, MA, USA) protein identification software by applying the search parameters previously reported [29]. Only the peptides with an expected value < 0.05 were considered. The assignment procedure was corroborated by the manual verification of the MS/MS spectra.

### 2.7. Identification and Quantification of Bioactive Peptides

The identification of the bioactive peptides with previously reported biological activity was achieved by using the Milk Bioactive Peptides Database (MBPDB) [37].

The relative amount of the bioactive peptides was calculated by integrating the area under the peak (AUP), measured from the extracted ion chromatograms (EIC) obtained for each peptide (tolerance ± 5 ppm). The data are expressed as AUP/mg of cheese. The anti-hypertensive peptides VPP and IPP as well as the DPP-IV-inhibitory peptides IPI and APFPE were quantified with parallel-reaction monitoring using pure peptide solutions as described in Martini et al. [13].

### 2.8. Statistical Analysis

The results are expressed as mean ± standard deviation (SD) for three replicates for every sample. ANOVA (univariate analysis of variance) analysis followed by the Tukey post-hoc test were executed using GraphPad Prism 6.0 (GraphPad Software, San Diego, CA, USA). The differences were considered significant when *p* < 0.05.

## 3. Results and Discussion

### 3.1. Chemical Composition and Peptide Quantification in the Six Different Cheese Varieties

The chemical composition of the different analysed cheeses is detailed in Table 2.

As can be seen, the various cheeses differed in their chemical composition. The main differences were related to the production process. The cooked cheeses (i.e., Ras, Gouda and Edam) showed significantly higher values of moisture, fat and proteins compared to the uncooked cheeses (Karish, Feta-type and Domiati). The lowest protein content was detected in Feta-type cheese, whereas Karish contained the lowest amount of fat being manufactured from skimmed buffalo milk.

**Figure 1:**
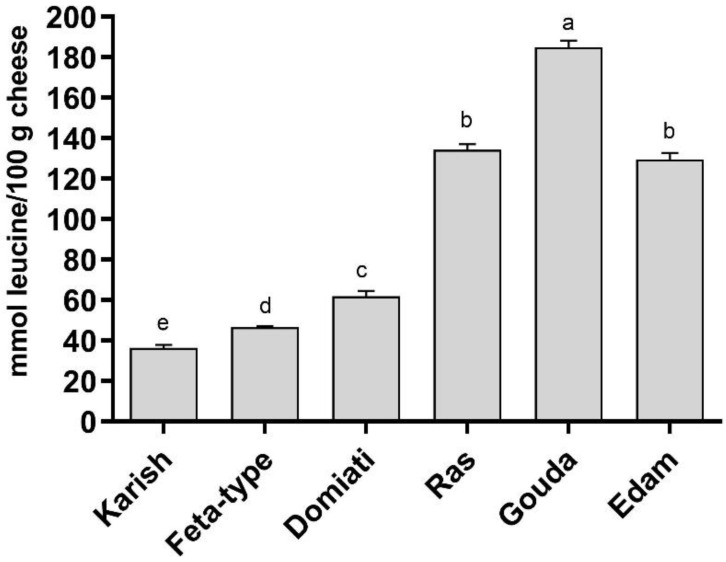
Total amount of low-molecular weight peptides (<3 kDa) in the different analysed cheese varieties. Values are the means of three assay replications ± standard deviation (SD). Different letters indicate significantly different values (*p* < 0.05).

A great variability among the cheeses was also observed when the total amount of low-molecular weight peptides (<3 kDa) was evaluated (Figure 1). The amount of low-molecular weight peptides was clearly related to the cooking and ripening procedure. The three cooked and ripened cheeses (Ras, Gouda and Edam) showed the highest amounts of peptides. In particular, Gouda cheese showed a significantly (*p* < 0.05) higher amount of peptides than Ras and Edam, whereas no significant differences were observed between Ras and Edam cheeses (*p* > 0.05). Domiati cheese, which was ripened but uncooked, displayed a significantly lower amount of peptides than the three ripened and cooked cheeses (*p* < 0.05) but still higher (*p* > 0.05) than the two non-ripened and uncooked cheeses (i.e., Feta-type and Karish). This last sample exhibited the lowest amount of low-molecular weight peptides (Figure 1). Finally, there was not an evident trend between the total amount of peptides and the total amount of proteins in the analysed cheeses.

Previous studies found a correlation between heat treatment and/or ripening and the amount of low-molecular weight peptides in cheese samples [13,15,38].

### 3.2. Biological Activities Assessment

**Figure 2:**
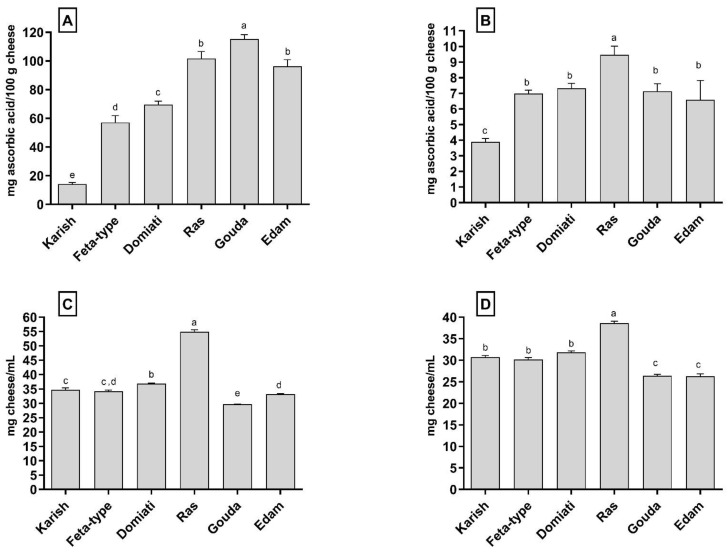
Biological activities of the cheese peptide fractions. (**A**) Antioxidant activity assayed with the ABTS method. (**B**) Antioxidant activity assayed with the FRAP method. (**C**) Angiotensin-converting enzyme (ACE) inhibitory activity. (**D**) Dipeptidyl-peptidase IV (DPP-IV) inhibitory activity. ACE inhibitory activity and DPP-IV-inhibitory activity were expressed as IC_50_, representing the cheese amount (expressed in mg of cheese/mL) able to inhibit the enzymatic activity by 50%. The IC_50_ values were calculated by plotting the cheese amount (base-10 logarithm) as a function of the percentage of inhibition. Values are the means of three assay replications ± standard deviation (SD). Different letters indicate significantly different values (*p* < 0.05).

The biological activities of the low-molecular weight peptide fractions extracted from the cheese samples were established with several in vitro assays aimed to assess the antioxidant, the ACE-inhibitory and the DPP-IV-inhibitory activities. To compare the biological profile of the analysed cheeses, the data are expressed as a function of the grams of cheese. In the antioxidant activity assays, the data are reported as mg of ascorbic acid equivalent referred to 100 g of cheese, which means that the higher the value, the greater the activity. In the ACE-inhibitory and DPP-IV-inhibitory assays, the data are shown as the IC_50_ values expressed as the amount of cheese in mg able to reduce the enzymatic activity by 50%. This means that for these assays, the lower the value the greater the activity. All the data are reported in Figure 2.

The antioxidant activity of the low-molecular weight peptide fractions extracted from the cheese samples was evaluated by using two different assays (ABTS and FRAP assays). As can be seen in Figure 2A, there were significant differences in the ABTS-scavenging ability of the peptides extracted from the different cheeses. The peptide fraction from Gouda cheese showed the highest activity (115.22 ± 3.12 mg of ascorbic acid/100 g of cheese) followed by the peptide fractions from Ras and Edam cheeses (101.80 ± 4.77 mg of ascorbic acid/100 g of cheese and 96.32 ± 4.51 mg of ascorbic acid/100 g of cheese, respectively). The Karish peptide fraction displayed the lowest ABTS-scavenging activity (14.11 ± 1.18 mg of ascorbic acid/100 g of cheese), whereas the Feta-type and Domiati peptide fractions exhibited intermediate values.

As can be observed by comparing the trends of the total amount of peptides (Figure 1) and the data reported in Figure 2A, it is possible to notice that there was a clear positive relationship between the total amount of peptides and the ABTS-scavenging activity, as previously suggested [39]. Moreover, ripening and especially cooking had positive effects on the ABTS-scavenging activity since the three cooked and ripened cheeses showed the highest values.

These relationships cannot be observed when the antioxidant activity was determined with the FRAP assay (Figure 2B). The Karish peptide fraction still showed the lowest activity (3.90 ± 0.21 mg of ascorbic acid/100 g of cheese); however, the highest activity was found for the Ras peptide fraction (9.46 ± 0.55 mg of ascorbic acid/100 g of cheese), and no significant differences (*p* > 0.05) were observed among the Feta-type, Domiati, Gouda and Edam peptide fractions.

The differences between the ABTS and FRAP assays may be related to the different reaction mechanisms. In the FRAP assay, single electron transfer (SET) is the predominant antioxidant mechanism, whereas in the ABTS assay, the antioxidant mechanism may involve both the single electron transfer (SET) and hydrogen atom transfer (HAT) [40]. For antioxidant peptides, the predominant antioxidant mechanism strongly depends on the presence of specific amino acids. For example, Y-containing antioxidant peptides acted mainly through a HAT-dependent mechanism, whereas C-containing antioxidant peptides acted mainly through a SET-dependent mechanism [41].

Previous studies found that water-soluble extracts from Gouda and Domiati cheeses showed antioxidant activities [42,43].

The ACE-inhibitory activity of the peptide fractions obtained from the different cheeses is reported in Figure 2C, and the results were expressed as mg of cheese per millilitre. Among the different samples, Gouda cheese displayed the highest ACE-inhibitory activity (*p* < 0.05) with an IC_50_ value of 29.76 ± 0.08 mg of cheese/mL. Vice versa, Ras cheese showed the lowest ACE-inhibitory potency (*p* < 0.05) with an IC_50_ value of 54.94 ± 0.73 mg of cheese/mL. The other four cheeses exhibited similar IC_50_ values in the range between 33.27 and 36.93 mg of cheese/mL. It is arduous to confront the ACE-inhibitory activity data obtained in different studies because of the application of distinct ACE assays and extraction protocols as well as the different ways that the data are expressed (such as the percentage of inhibition or IC_50_ values calculated on the basis of the protein or peptide content).

Butikofer et al. [16] analysed 44 traditional cheeses for their ACE-inhibitory activity and expressed the results as mg of cheese in the test solution. They found that the IC_50_ values ranged between 2.0 and 29.5 mg of cheese/mL depending on the cheese type. In particular, the authors measured the IC_50_ values of Gouda, Edam and Feta cheeses that resulted in values of 2.0, 13.3 and 14.3 mg of cheese/mL, respectively. In another study, the IC_50_ value of a water-soluble extract from Emmenthal cheese was found to be 27.6 mg of cheese/mL [44]. Despite the same trend of ACE-inhibitory activity (i.e., Gouda more active than Edam and Feta), the data reported in this study were higher than the previously reported data. This discrepancy may be due to the different assays used in the previous investigations and in this study, which used different substrates, such as Hippuryl-His-Leu-OH (HHL assay) and FAPGG (FAPGG assay), respectively. In this sense, it is well-known that the inhibitory activity of peptides is strongly substrate-dependent with the FAPGG assay giving higher IC_50_ values than the HHL assay [45]. This substrate-dependence is related to the different binding affinity of FAPGG and HLL versus ACE with the last substrate having a Km value approximately ten-times higher with respect to FAPGG [46].

Finally, no clear relationships were found between the ACE-inhibitory activity and the total amount of peptides, cheese cooking or ripening.

When the DPP-IV-inhibitory activity of the different peptide fractions extracted from the cheeses was assessed, Gouda and Edam cheeses were still the most active, showing no statistically different (*p* > 0.05) IC_50_ values (Figure 2D). Once again, Ras cheese showed the lowest inhibitory activity, whereas no statistically significant differences (*p* > 0.05) were found for Karish, Feta-type and Domiati cheeses. One previous study determined the DPP-IV-inhibitory activity of a water-soluble extract from Gouda cheese [47], whereas no data in the literature are present concerning the other cheeses.

Also, in the case of the DPP-IV-inhibitory activity, no association was found with cheese cooking and ripening and with the peptide concentration.

In the α-amylase activity assay, only the peptide fractions extracted from Karish and Feta-type cheeses were able to inhibit the enzymatic activity with IC_50_ values of 3.09 ± 0.17 and 3.10 ± 0.04 mg of cheese/mL, respectively. These results suggested that cooking and especially ripening had a negative effect on the α-amylase-inhibitory activity of the cheeses. Previous studies found that the water-soluble extracts from Prato, Parmigiano Reggiano, Akawi and Himalayan cheeses were able to inhibit α-amylase activity [29].

Finally, no α-glucosidase-inhibitory activity was detected in any cheese peptide fractions.

### 3.3. General Peptidomics Profiles

**Figure 3:**
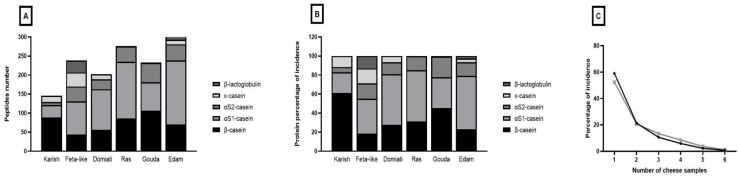
The peptidomics analysis of the cheese low-molecular weight peptide fractions. (**A**) Number of identified peptides divided by protein type. (**B**) Protein percentage distribution of peptides identified in the different cheeses. (**C**) Frequency of distribution of the number of peptides (black line) and of the number of bioactive peptides (grey line) identified in the different cheeses expressed as the percentage of incidence. The complete list of peptides identified in the different cheeses is reported in Supplementary Table S1.

The peptidomics analysis carried out with high-resolution mass spectrometry allowed us to identify a total of 809 peptides originating from the major milk proteins. The list of the identified peptides together with the mass spectrometry data are reported in supplementary Table S1. The highest number of identified peptides were derived from αS1-casein (307 peptides), β-casein (256 peptides) and αS2-casein (148 peptides), whereas only 63 and 35 peptides were derived from κ-casein and β-lactoglobulin, respectively. Regarding the different cheeses, the highest number of peptides was observed in Edam and Ras cheeses (301 and 276 peptides, respectively), whereas Karish displayed the lowest amount of identified peptides (Figure 3A). The peptide fraction of this last cheese was particularly enriched in β-casein-derived peptides that accounted for the 61.0% of total peptides (Figure 3A,B).

The only other cheese with a higher incidence of β-casein-derived peptides than other proteins was Gouda, which also displayed the greatest number of αS2-casein-derived peptides (Figure 3A,B). All the other cheeses’ peptide fractions, and especially from Edam, Ras and Domiati, were characterized for a prevalence of αS1-casein-derived peptides (Figure 3A,B). The peptides derived from κ-casein were generally identified in small numbers with respect to the other caseins with only one peptide identified in Ras and Gouda. The β-lactoglobulin-derived peptides were detected in only two samples (Feta-type and Edam peptide fractions) with Feta-type cheese displaying the highest number of β-lactoglobulin-derived peptides, where they accounted for 13.0% of the total peptides.

The analysis of the peptide frequency (Figure 3C) revealed that most of the identified peptides were detected in just one cheese sample (59.1% of total peptides, corresponding to 478 peptides), whereas only eighteen and six peptides (corresponding to the 2.2% and 0.7% of total peptides) were ascertained in five and six cheese samples, respectively. These results proved the great diversity in the peptide profiles of the different cheeses studied. This diversity may be related to the different manufacturing conditions to which the cheeses were subjected.

### 3.4. Bioactive Peptide Profiles

Considering all the cheese samples, a total of 82 peptides were classified as bioactive peptides sharing 100% of homology with previously reported biologically active peptides (Table 3).

The most represented bioactivities were ACE-inhibitory, antioxidant and DPP-IV-inhibitory activities. In particular, 47 peptides showed ACE-inhibitory activity, 26 peptides displayed antioxidant properties and 15 peptides were DPP-IV-inhibitors. Moreover, thirteen peptides were anti-microbial, five showed opioid activity, four were anti-inflammatory, three were immunomodulators, and two peptides displayed anxiolytic and anti-cancer activities. In addition, 22 peptides exhibited two or more functionalities and can be classified as multi-functional peptides.

Looking at the individual cheese samples, the highest number of bioactive peptides were found in Gouda (37 bioactive peptides) and Edam (33 bioactive peptides), whereas the lowest amount was found in Karish (19 bioactive peptides). Gouda and Edam cheeses also displayed the highest number of ACE-inhibitory and DPP-IV-inhibitory peptides, whereas the highest number of antioxidant peptides was found in Edam and Karish. No DPP-IV-inhibitory peptides were identified in Feta-type cheese.

As reported in Figure 3C, the distribution frequency of the bioactive peptides among the cheese samples demonstrated that most of the bioactive peptides were found in only one cheese sample (52.4% of the total bioactivity peptides, corresponding to 43 bioactive peptides). Only one peptide, the αS1-casein-derived ACE-inhibitory peptide ENLLRF, was identified in all six cheese samples, whereas the antioxidant peptide YLG as well as the ACE-inhibitory peptides VPP and IPP were detected in five cheese samples (Karish, Domiati, Ras, Gouda and Edam). The highest diversity was observed in Feta-type cheese with 13 unique bioactive peptides detected exclusively in this sample and corresponding to the 61.9% of total bioactive peptides identified in Feta-type cheese.

#### 3.4.1. Antioxidant Peptides

A total of 26 peptides with previously reported antioxidant properties were identified considering all the cheese samples (Table 3). The majority of identified antioxidant peptides contained the antioxidant amino acid Y in their sequence. Previous studies highlighted that the occurrence of an antioxidant amino acid is essential for the antioxidant ability of peptides [41,48,49]. The highest number of antioxidant peptides was identified in Edam and Karish with 12 and 10 detected antioxidant peptides, respectively.

Several identified antioxidant peptides contained repetitive structural motifs that conferred the antioxidant properties to the peptides. Six antioxidant peptides contained the structural motif YLG (or LGY) in their sequence and presented high ABTS-scavenging activity. The relative quantification of these peptides demonstrated that Edam is particularly rich in the αS1-casein-derived antioxidant peptides RYLGY, RYLGYLE, YLGYLE, YLGY and YLG that can be responsible for the high ABTS-scavenging activity of this cheese [50] (Table S2). In addition, some of these peptides showed a relative high amount in Domiati (RYLGYLE, YLGYLE, YLG and LGY) and Feta-type (RYLGY and LGY) cheeses. Five β-casein-derived antioxidant peptides contained the structural motif PYPQ, which is primary in determining the antioxidant properties of these peptides [51,52]. Some of these peptides displayed a relatively high amount in Edam (VPYPQ, VPYPQR and PYPQ) and Gouda (VPYPQ) cheeses. Furthermore, Gouda cheese was the only sample containing the ABTS-radical scavenging peptides YPFPGPI, YPFPGPIN and FPQY.

The β-casein-derived antioxidant peptide LLY, identified in Karish, Gouda and Edam cheeses, exhibited in vivo antioxidant properties in mice after ethanol-induced oxidative stress [53].

#### 3.4.2. ACE-Inhibitory Peptides

A total of 47 peptides with previously reported ACE-inhibitory activity were detected considering all the cheese samples (Table 3). Most of these peptides were released from β-casein (26 ACE-inhibitory peptides) and αS1-casein (11 ACE-inhibitory peptides). The remaining ACE-inhibitory peptides were from αS2-casein (five ACE-inhibitory peptides), κ-casein (three ACE-inhibitory peptides) and β-lactoglobulin (two ACE-inhibitory peptides). The highest number of ACE-inhibitory peptides was found in Edam, Gouda and Ras cheeses with 19, 18 and 17 ACE-inhibitory peptides identified, respectively.

Some of the identified peptides also showed in vivo anti-hypertensive activity beyond the ACE-inhibitory one. The two lactotripeptides VPP and IPP, identified in all the cheese samples apart from Feta-type cheese, have been extensively studied for their anti-hypertensive effect in human subjects [54,55,56,57]. A recent meta-analysis of clinical trials highlighted an average reduction on systolic blood pressure of 3.73 mmHg and 2.95 mmHg in pre-hypertensive and hypertensive human subjects, respectively, at doses from 3 to 10 mg/day [54,55].

Several other peptides identified in the cheeses have been reported as anti-hypertensive in spontaneously hypertensive rats (SHR) (Table 3). The β-casein-derived peptides KVLPVPQ (identified in Feta-type and Edam) and LHLPLP (identified in Karish) as well as the αS1-casein-derived peptide RYLGY (identified in Feta-type and Edam) displayed potent anti-hypertensive activity, reducing the systolic blood pressure in SHR of 31.5, 25.3 and 25 mmHg, respectively [12].

The anti-hypertensive peptides VPP and IPP were quantified in the different samples (Table 4).

The highest amount of both VPP and IPP was found in Gouda cheese (39.2 ± 2.3 and 17.7 ± 1.1 mg/100 g, respectively), which also showed the highest ACE-inhibitory activity, followed by Domiati (3.0 ± 0.1 and 8.4 ± 0.4 mg/100 g of VPP and IPP, respectively) and Edam (3.7 ± 0.3 and 3.3 ± 0.1 mg/100 g of VPP and IPP, respectively) cheeses. Considering the above reported doses for the observed anti-hypertensive effect of VPP and IPP and based on their concentration in the cheeses, it is possible to expect an effect in pre-hypertensive and hypertensive subjects after consumption of 10–20 g of Gouda cheese, 50–100 g of Domiati and 100 g of Edam.

The other ACE-inhibitory peptides were relatively quantified in the cheese samples (Table S2). Despite being the richest in VPP and IPP and exerting the highest ACE-inhibitory activity, Gouda cheese was also the richest in the potent ACE-inhibitory peptides RPKHPIKHQ, YPFPGPIPN and RPKHPI. The second most potent ACE-inhibitory sample was Edam cheese. Similar to Gouda, this cheese contained the ACE-inhibitory peptides VPP, IPP, RPKHPIKHQ and RPKHPI and was the richest in the potent ACE-inhibitory peptide RYLGY (also showing an in vivo anti-hypertensive effect in SHR) as well as in the peptides YLGY, VRYL and DERF. Feta-type cheese did not contain the peptides VPP and IPP but was rich in the peptide RYLGY as well as in the ACE-inhibitory peptides LGY, AIPPKKNQD and DAQSAPLRVY, which can be responsible for the ACE-inhibitory activity of this cheese. Instead, Domiati cheese contained both the tripeptides VPP and IPP as well as the potent ACE-inhibitory peptides RPKHPI, LGY and DERF and was the richest in IQA. Karish cheese contained a low amount of VPP and IPP but was the only one having the potent ACE-inhibitory peptides LHLPLP (that also exhibited high in vivo anti-hypertensive activity in SHR), NLHLPLPLL and NLHLPLP. Finally, Ras was the only cheese containing the peptides AVPYPQR and ITP as well as low amounts of VPP, IPP, RPKHPI and YLGY.

#### 3.4.3. DPP-IV-Inhibitory Peptides

A total of 15 peptides previously identified as DPP-IV-inhibitors were detected in the cheese samples (Table 3). The highest number of these bioactive peptides was observed in Gouda (fourteen peptides) and Edam (seven peptides) that also showed the highest DPP-IV-inhibitory activity.

Uenishi et al. [47] characterized 10 DPP-IV-inhibitory peptides in commercial Gouda-type cheeses. A total of six of these peptides (YPFPGPIPN, FPGPIPN, LPQNIPP, PQNIPPL, VPITPT and VPITPTL) were present also in the Gouda cheese analysed in this study. The most potent DPP-IV-inhibitory peptide was IPI, identified only in the Edam sample. This peptide was quantified resulting in an amount of 1.4 ± 0.1 mg/100 g of cheese (Table 4). In addition, the potent αS1-casein-derived DPP-IV-inhibitory peptide APFPE, previously identified for the first time in Parmigiano Reggiano cheese [29], was also quantified (Table 4). This peptide was present in Domiati, Ras, Gouda and Edam, and the highest concentrations were found in the last two cheese samples (509.1 ± 21.2 and 298.0 ± 13.6 mg/100 g of Edam and Gouda, respectively).

The relative quantification analysis of two additional peptides with high DPP-IV-inhibitory activity, LPVPQ and VPYPQ [58,59], suggested the presence of higher amounts of these peptides in Gouda and Edam cheese that also evidenced the highest DPP-IV-inhibitory activity (Table S2 and Figure 2). Finally, no peptides with previously reported DPP-IV-inhibitory activity were detected in Feta-type-cheese.

## 4. Conclusions

The low-molecular weight peptide fractions from three traditional Egyptian cheeses (Karish, Domiati and Ras) and three cheese varieties consumed worldwide (Feta-type, Gouda and Edam) were characterized for their content in bioactive peptides and for their biological activities. All the cheeses displayed antioxidant, ACE-inhibitory and DPP-IV-inhibitory activities with some differences. Gouda cheese exhibited the highest activity in all the assays.

A total of 809 peptides were identified in the analysed cheeses with most of them found in only one cheese sample. The differences in the technological procedures, manufacturing conditions, ripening time and starter and non-starter lactic acid bacteria determined a great variability in the peptide profiles of the cheeses. A total of 82 bioactive peptides, mainly ACE-inhibitory, DPP-IV-inhibitory and antioxidant peptides, were identified and, once again, most of these peptides were detected in only one cheese sample.

The anti-hypertensive lactotripeptides VPP and IPP with proven in vivo effects in humans as well as the DPP-IV-inhibitory peptides IPI and APFPE were quantified.

Gouda cheese was an especially good source of VPP and IPP, present in concentrations compatible with an anti-hypertensive effect after the intake of a portion of cheese. Moreover, Gouda and Edam cheeses also contained considerable amounts of the DPP-IV-inhibitory peptide APFPE, suggesting that this peptide can reach intestinal concentrations similar to the IC_50_ value against DPP-IV after the consumption of a portion of these cheeses.

Although further in vivo investigations are needed, the results of this study identified a possible role in the prevention of cardiovascular diseases and diabetic complications of Gouda and Edam cheeses. In any case, the reported data lay the basis for further in vivo exploration of the health benefits associated with the intake of some of these cheeses.

## Figures and Tables

**Table 1:** Main manufacturing conditions and characteristics of the six different cheese varieties analysed in this study.

Cheese Variety	Milk ^1^	Starter ^2^	Rennet	Cooking	Ripening (Days)
Karish	B, P	F	N	-	-
Feta-type	C, P	N	Yes	-	-
Domiati	C, R	N	Yes	-	90
Ras	C, R	N	Yes	45 °C/45 min	90
Gouda	C, P	F	Yes	38 °C/45 min	90
Edam	C, P	F	Yes	36 °C/40 min	90

^1^ C: cow; B: buffalo; P: pasteurized; R: raw. ^2^ F: freeze-dried; N: none.

**Table 2:** Gross chemical composition of the different types of cheese.

Cheese Variety	Moisture (%)	Fat (%)	Protein (%)	Salt (%)
Karish	69.04 ± 3.35 ^a^	0.55 ± 0.05 ^e^	17.59 ± 1.01 ^b^	2.47 ± 0.14 ^c^
Feta-type	59.32 ± 2.11 ^b^	20.34 ± 0.98 ^d^	9.11 ± 0.88 ^c^	3.23 ± 0.29 ^b^
Domiati	53.60 ± 2.28 ^c^	23.98 ± 0.77 ^c^	16.37 ± 0.53 ^b^	4.02 ± 0.17 ^a^
Ras	34.90 ± 2.09 ^e^	34.30 ± 1.95 ^a^	25.90 ± 1.67 ^a^	3.21 ± 0.19 ^b^
Gouda	38.20 ± 2.18 ^e^	33.18 ± 1.66 ^a^	26.10 ± 1.34 ^a^	2.00 ± 0.17 ^d^
Edam	44.15 ± 2.23 ^d^	27.22 ± 1.84 ^b^	25.17 ± 1.75 ^a^	1.82 ± 0.15 ^d^

Different letters in the same column indicate significant differences (*p <* 0.05); n = 3.

**Table 3:** Peptides sharing 100% of sequence homology with previously reported bioactive peptides identified in Karish (K), Feta-type (F), Domiati (D), Ras (R), Gouda (G) and Edam (E) cheeses.

Peptide Sequence	Protein Fragment	Bioactivity	Sample
RPKHPI	αS1-casein (1–6)	ACE-inhibitor	D, R, G and E
RPKHPIK	αS1-casein (1–7)	Anti-microbial	G
RPKHPIKHQ	αS1-casein (1–9)	ACE-inhibitor, anti-hypertensive in vivo	G and E
VLNENLLR	αS1-casein (15–22)	Anti-microbial	D, R, G and E
ENLLRF	αS1-casein (18–23)	ACE-inhibitor	K, F, D, R, G and E
LRFF	αS1-casein (19–24)	ACE-inhibitor	F
FVAPFPEVFG	αS1-casein (24–33)	ACE-inhibitor	D and R
APFPE	αS1-casein (26–30)	DPP-IV-inhibitor	D, R, G and E
PFP	αS1-casein (27–29) β-casein (61–63)	ACE-inhibitor	R
FPEVFGK	αS1-casein (28–34)	ACE-inhibitor	D, R, G and E
RYLGY	αS1-casein (90–94)	ACE-inhibitor, anti-hypertensive in vivo, antioxidant, opioid	F and E
RYLGYLE	αS1-casein (90–96)	Antioxidant, anti-cancer	D and E
YLG	αS1-casein (91–93)	Antioxidant	K, D, R, G and E
YLGY	αS1-casein (91–94)	ACE-inhibitor, antioxidant	R and E
YLGYLE	αS1-casein (91–96)	ACE-inhibitor, antioxidant, opioid	D, R and E
LGY	αS1-casein (92–94)	ACE-inhibitor, antioxidant	K, F and D
YLEQLLR	αS1-casein (94–100)	Anti-microbial	R
LRLKKYKVPQL	αS1-casein (99–109)	Anti-microbial	F
YFYPE	αS1-casein (144–148)	Opioid	E
FYPEL	αS1-casein (145–149)	ACE-inhibitor, antioxidant	F
PEL	αS1-casein (147–149)	Antioxidant	F and R
APSFSDIPNPIGSENSE	αS1-casein (176–192)	Antioxidant	F
YQKFPQY	αS2-casein (89–95)	ACE-inhibitor, antioxidant	F
FPQY	αS2-casein (92–95)	ACE-inhibitor, antioxidant	G
VPITPT	αS2-casein (117–122)	DPP-IV-inhibitor	R and G
VPITPTL	αS2-casein (117–123)	DPP-IV-inhibitor	G
ITP	αS2-casein (119–121)	ACE-inhibitor	R
LKKISQ	αS2-casein (164–169)	Anti-microbial	F
VYQHQKAMKPWIQPKTKVIPYVRYL	αS2-casein (183–207)	Anti-microbial	G
IQPKTKVIPYVR	αS2-casein (194–205)	Anti-microbial	D and G
TKVIP	αS2-casein (198–202)	ACE-inhibitor, anti-hypertensive in vivo	G
TKVIPYVRYL	αS2-casein (198–207)	Anti-microbial	G and E
VRYL	αS2-casein (204–207)	ACE-inhibitor	E
RELEEL	β-casein (1–6)	Antioxidant	F, G and E
VPGEIVE	β-casein (8–14)	DPP-IV-inhibitor	D, R, G and E
YPFPGP	β-casein (60–65)	DPP-IV-inhibitor, opioid	G
YPFPGPI	β-casein (60–66)	DPP-IV-inhibitor, antioxidant, opioid, immunomodulator, anxiolytic, anti-cancer	G
YPFPGPIPN	β-casein (60–68)	ACE-inhibitor, anti-hypertensive in vivo, DPP-IV-inhibitor, antioxidant	G
FPGPIPN	β-casein (62–68)	DPP-IV-inhibitor	G
SLPQ	β-casein (69–72)	ACE-inhibitor	R
LPQNIPP	β-casein (70–76)	DPP-IV-inhibitor	G
PQNIPPL	β-casein (71–77)	DPP-IV-inhibitor	G
IPP	β-casein (74–76) κ-casein (108–110)	ACE-inhibitor, anti-hypertensive in vivo, DPP-IV-inhibitor, antioxidant, anti-inflammatory	K, D, R, G and E
TPVVVPPFLQP	β-casein (80–90)	ACE-inhibitor, anti-hypertensive in vivo	G
PVVVPPFLQPE	β-casein (81–91)	Anti-microbial	F
VVPP	β-casein (83–86)	ACE-inhibitor	R and G
VPP	β-casein (84–86)	ACE-inhibitor, anti-hypertensive in vivo, antioxidant, anti-inflammatory	K, D, R, G and E
FPKYPVEPF	β-casein (111–119)	Antioxidant	D and R
NLHLPLP	β-casein (132–138)	ACE-inhibitor	K
NLHLPLPLL	β-casein (132–140)	ACE-inhibitor	K
LHLPLP	β-casein (133–138)	ACE-inhibitor, anti-hypertensive in vivo	K
LHLPLPL	β-casein (133–139)	ACE-inhibitor	F
LPLP	β-casein (135–138)	ACE-inhibitor, anti-hypertensive in vivo	R, G and E
LPLPL	β-casein (135–139)	DPP-IV-inhibitor	G
LPLPLL	β-casein (135–140)	ACE-inhibitor	G
SQSKVLPVPQ	β-casein (166–175)	ACE-inhibitor	K
SQSKVLPVPQKAVPYPQ	β-casein (166–182)	Antioxidant	F
KVLPVPQ	β-casein (169–175)	ACE-inhibitor, anti-hypertensive in vivo, anti-inflammatory	F and E
LPVPQ	β-casein (171–175)	DPP-IV-inhibitor	D, R, G and E
AVPYPQR	β-casein (177–183)	ACE-inhibitor, anti-hypertensive in vivo, antioxidant, anti-microbial	R
VPYPQ	β-casein (178–182)	DPP-IV-inhibitor, antioxidant	F, G and E
VPYPQR	β-casein (178–183)	Antioxidant	E
PYPQ	β-casein (179–182)	Antioxidant	K and E
RDMPIQAF	β-casein (183–190)	ACE-inhibitor	K and D
IQA	β-casein (187–189)	ACE-inhibitor	K, F and D
LLY	β-casein (191–193)	Antioxidant, anti-inflammatory, immunomodulator	K, G and E
YQEP	β-casein (193–196)	ACE-inhibitor, antioxidant	K
YQEPVLGP	β-casein (193–200)	ACE-inhibitor, antioxidant	K
YQEPVLGPVRGPFPIIV	β-casein (193–209)	ACE-inhibitor, anti-microbial, immunomodulator	R and E
EPVLGPVRGPFP	β-casein (195–206)	ACE-inhibitor	G and E
VLGP	β-casein (197–200)	ACE-inhibitor, DPP-IV-inhibitor	K, D, G and E
VRGPFP	β-casein (201–206)	ACE-inhibitor	R, G and E
VRGPFPIIV	β-casein (201–209)	ACE-inhibitor, anti-hypertensive in vivo	R, G and E
DERF	κ-casein (14–17)	ACE-inhibitor	D and E
IPI	κ-casein (26–28)	DPP-IV-inhibitor	E
YVL	κ-casein (30–32)	Antioxidant, anti-microbial	K, F and D
YGL	κ-casein (38–40)	ACE-inhibitor	K and D
AIPPKKNQD	κ-casein (107–115)	ACE-inhibitor	F
EIPT	κ-casein (118–121)	Anti-microbial	D
LDAQSAPLR	β-lactoglobulin (32–40)	ACE-inhibitor	F
DAQSAPLRVY	β-lactoglobulin (33–42)	ACE-inhibitor	F
HIRL	β-lactoglobulin (146–149)	Anxiolytic	F

Peptide bioactivities were obtained from the Milk Bioactive Peptides Database. Abbreviations: ACE: angiotensin-converting enzyme; DPP-IV: dipeptidyl peptidase IV.

**Table 4:** The amounts of bioactive peptides in the low-molecular weight peptide fractions of the analysed cheeses. The results are reported as mg/100 g of cheese.

Sequence	Karish	Feta-Type	Domiati	Ras	Gouda	Edam
VPP ^a^	0.94 ± 0.05 ^e^	n.d.	3.03 ± 0.11 ^c^	0.68 ± 0.03 ^d^	39.19 ± 1.26 ^a^	3.73 ± 0.17 ^b^
IPP ^a^	0.83 ± 0.05 ^e^	n.d.	8.37 ± 0.48 ^b^	2.50 ± 0.10 ^d^	17.72 ± 0.89 ^a^	3.29 ± 0.18 ^c^
APFPE ^b^	n.d.	n.d.	17.20 ± 0.94 ^c^	3.27 ± 0.29 ^d^	298.02 ± 15.36 ^b^	509.13 ± 20.44 ^a^
IPI ^b^	n.d.	n.d.	n.d.	n.d.	n.d.	1.38 ± 0.09

^a^ ACE-inhibitory and anti-hypertensive peptides. ^b^ DPP-IV-inhibitory. n.d. means peptide not detected in the sample. Different letters in the same row indicate significantly different values (*p* < 0.05).

## Data Availability

The data presented in this study are available herein.

## Supplementary Materials

The following supporting information can be downloaded at: https://www.mdpi.com/article/10.3390/biology12010078/s1, Table S1: Peptide profiles of Karish, Feta-type, Domiati, Ras, Gouda and Edam low-molecular weight peptide fractions; Table S2: Relative quantification of the ACE-inhibitory, DPP-IV-inhibitory and antioxidant peptides identified in the low-molecular weight peptide fractions of the analysed cheeses.
